# A Holistic View of Advanced Heart Failure

**DOI:** 10.3390/life12091298

**Published:** 2022-08-24

**Authors:** Filippos Triposkiadis, Grigorios Giamouzis, Takeshi Kitai, John Skoularigis, Randall C. Starling, Andrew Xanthopoulos

**Affiliations:** 1 Department of Cardiology, University Hospital of Larissa, 411 10 Larissa, Greece; 2 National Cerebral and Cardiovascular Center, Osaka 564-8565, Japan; 3 Kaufman Center for Heart Failure Treatment and Recovery, Heart, Vascular, and Thoracic Institute, Cleveland, OH 44195, USA

**Keywords:** advanced heart failure, mechanical circulatory support, heart transplantation, shock

## Abstract

Advanced heart failure (HF) may occur at any level of left ventricular (LV) ejection fraction (LVEF). The latter, which is widely utilized for the evaluation of LV systolic performance and treatment guidance of HF patients, is heavily influenced by LV size and geometry. As the accurate evaluation of ventricular systolic function and size is crucial in patients with advanced HF, the LVEF should be supplemented or even replaced by more specific indices of LV function such as the systolic strain and cardiac power output and size such as the LV diastolic diameters and volumes. Conventional treatment (cause eradication, medications, devices) is often poorly tolerated and fails and advanced treatment (mechanical circulatory support [MCS], heart transplantation [HTx]) is required. The effectiveness of MCS is heavily dependent on heart size, whereas HTx which is effective in the vast majority of the cases is limited by the small donor pool. Expanding the MCS indications to include patients with small ventricles as well as the HTx donor pool are major challenges in the management of advanced HF.

## 1. Introduction

Heart failure (HF) is a chronic evolving syndrome. Although a lot of patients respond to treatment some patients with HF develop advanced HF characterized by increased morbidity and mortality risk which increases with each subsequent HF hospitalization [1,2,3]. Several classification systems have been proposed to define those subjects with advanced HF. An easy to memorize definition, the ABCDEFGH definition of advanced HF, based on the position statements of the Heart Failure Society of America and the Heart Failure Association of the European Society of Cardiology is presented in Table 1 [4,5].

In addition, the Interagency Registry for Mechanically Assisted Circulation (INTERMACS) classification system was developed to risk stratify patients with advanced HF (Table 2) [6].

**Figure 1:**
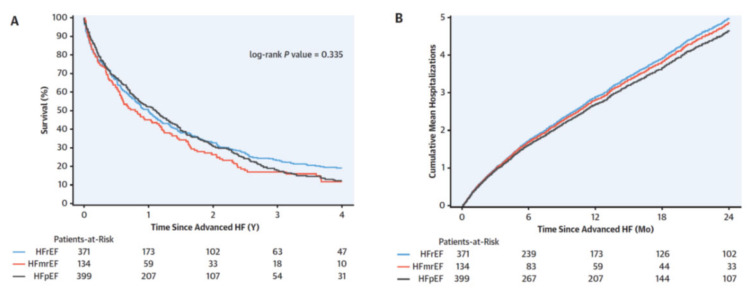
Survival and hospitalizations after advanced heart failure (HF). (**A**) The Kaplan-Meier survival curves according to ejection fraction. (**B**) Mean cumulative hospitalizations after advanced heart failure according to ejection fraction. HFmrEF = heart failure with mid-range ejection fraction; HFpEF = heart failure with preserved ejection fraction; HFrEF = heart failure with reduced ejection fraction. Adapted with permission from Ref. [7]. Copyright © 2022 by the American College of Cardiology Foundation. Published by Elsevier.

Advanced HF may occur at any level of left ventricular (LV) ejection fraction (LVEF). In a recent study including 936 patients with advanced HF, 42.3% had HF with reduced (<40%) LVEF (HFrEF), 14.3% HF with midrange (40–49%) LVEF (HFmrEF), and 43.4% had HF with preserved (≥50%) LVEF (HFpEF) [7]. Most importantly death from all-causes was similar in patients with HFrEF, HFmrEF or HFpEF (Figure 1) [7]. Likewise, in another retrospective cohort study of Olmsted County, Minnesota, which included 4,597 residents with incident HF, the cumulative incidence of advanced HF was 11.5% at 6 years after incident HF diagnosis overall. No significant difference in the risk of developing advanced HF in patients with incident HFrEF, HFmrEF, and HFpEF was observed [8].

This paper attempts a holistic approach of advanced HF, emphasizing that the use of LVEF as a diagnostic and treatment guide in this subgroup of HF patients may be misleading regarding pathophysiology, diagnosis, and management.

## 2. Pathophysiology

**Figure 2:**
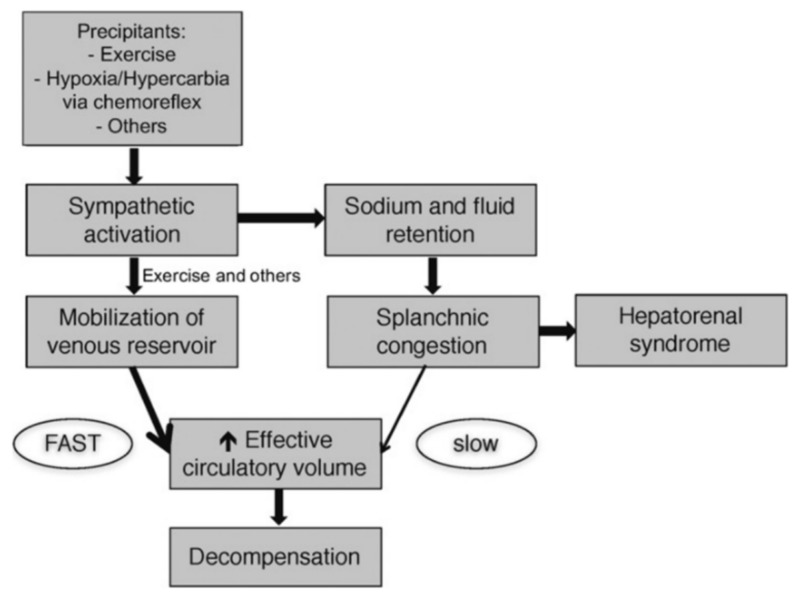
The proposed mechanism of progression from chronic compensated to acute heart failure is summarized in this figure. Sodium retention and fluid expansion result in an increase in unstressed volume and subsequent splanchnic congestion. This process is slow and takes days to weeks. The fast component often observed in the few days before decompensation is driven by autonomic imbalance with overactivity of the sympathetic nervous system. This results in an intercompartmental fluid shift into the central circulation with a subsequent accelerated increase in central filling pressures. Rapid fluid mobilization also occurs with activity and can explain exercise limitations experienced by heart failure patients. Adapted with permission from Ref. [12]. Copyright © 2022. Published on behalf of the American Heart Association, Inc., by Wiley.

The pathophysiologic mechanisms operating at the advanced stage of HF are virtually similar with those involved at the earlier stages of the disease, with the potential differences being quantitative rather that qualitative [9,10]. Fluid overload with central and/or peripheral congestion is a typical feature of advanced HF regardless of the LVEF and it is the main reason for hospital admission. The pathophysiology of congestion is complicated, and the hypothesis of intravascular fluid accumulation is insufficient [11]. The features of interstitial and intravascular fluid compartment interactions and fluid redistribution from venous splanchnic beds to central pulmonary circulation should also be considered (Figure 2) [12].

Orthostatic stress is a simple test that can be used for the evaluation of splanchnic vascular capacitance. The existence of orthostatic symptoms in a HF patient indicates normal to high splanchnic (and peripheral) capacitance, whereas the absence of orthostatic symptoms suggests the opposite [12]. Venous congestion has been reported to be the main hemodynamic factor leading to renal function impairment in patients with HF [13]. Congestive nephropathy is a potentially reversible subtype of renal dysfunction associated with decreasing renal venous outflow and escalating renal interstitial pressure [14]. Additionally, it has recently been proposed that the space between the kidney and its capsule may be limited in HF either by the rigid renal capsule that encloses the renal interstitial tissue or by the layer of fat around the kidneys or by the peritoneal space exerting pressure on the retroperitoneal kidneys leading to “renal tamponade” due to compression of the kidney caused by the limited space for expansion [15]. Similarly, venous congestion leads to hepatic dysfunction [16]. If long-standing, both congestive nephropathy and hepatopathy result in structural and functional alterations. Right ventricular (RV) dysfunction and failure are common in advanced HF, usually due to pulmonary hypertension [17,18]. Eventually venous congestion may end up to a vicious cycle of neurohormonal activation, increased intra-abdominal pressure, excessive renal tubular sodium reabsorption, and diuretic resistance leading to further right ventricular (RV) stress [14].

Advanced HF may occasionally progress to cardiogenic shock characterized by the presence of hypotension (systolic blood pressure [SBP] ≤ 90 mm Hg for at least 30 min or the need for supportive measures to maintain an SBP ≥ 90 mm Hg, or a drop in mean arterial blood pressure [MAP] > 30 mm Hg below the baseline), severely impaired cardiac output [cardiac index ≤ 1.8 L/min/m^2^ without support or <2.2 L/min/m^2^ with support] and increased pulmonary capillary wedge pressure ([PCWP] ≥15 mm Hg) [19,20]. End organ hypoperfusion in this setting results in a further deterioration of the usually preexisting hepatic and renal dysfunction, lactic acidemia, decreased coronary perfusion pressure, and further activation of baroceptors and chemoreceptors, all of which lead to a vicious circle of worsening cardiac performance [21]. If cardiogenic shock continues, a state of systemic inflammatory response syndrome ensues, and HF escalates from an initial hemodynamic disorder into a multisystem disorder [22,23].

## 3. Clinical Manifestations

Advanced HF is accompanied by progressive and/or persistent severe signs and symptoms of HF despite optimal (maximum tolerated doses up to target) evidence-based medical therapy, along with implantation of all appropriate devices (e.g., cardiac synchronization therapy) and with all reversible causes of HF managed (4). Advanced HF patients often suffer from severe physical and psychosocial symptoms and especially in the elderly, advanced HF usually develops along with other chronic diseases, resulting in complex coexisting morbidity [24]. Common clinical manifestations are virtually unrelated to LVEF [25] and include exercise intolerance, unintentional weight loss, refractory volume overload, recurrent ventricular arrhythmias, as well as hypotension and signs of poor perfusion. Patients with advanced HF generally are in New York Heart Association (NYHA) functional class III (symptoms with minimal exertion) or IV (symptoms at rest or with any activity). Severe limitation of exercise ability is one of the cardinal manifestations of advanced HF. However, as exercise capacity may vary depending on individual characteristics (e.g., age and activity level), querying a patient for changes in exercise capacity over time can be most informative. Exercise limitation that should be considered worrisome for advanced HF includes the inability to walk a city block or perform daily activities such as bathing, shaving, or dressing in the absence of limiting symptoms [1]. Many patients will end up having dyspnea at rest (orthopnea, paroxysmal nocturnal dyspnea). Poor functional status is a marker of unfavorable prognosis in patients with HF [26]. The diagnosis of cardiogenic shock can sometimes be made at the bedside and is characterized by the presence of low blood pressure in the absence of hypovolemia as well as clinical signs of inadequate tissue perfusion (e.g., cyanosis, cool extremities, oliguria, and altered mentation).

## 4. Evaluation of Ventricular Systolic Function

Evaluation of LV systolic function is important in advanced HF. The LVEF is inappropriately used as a measure of LV systolic function in this context, whereas other noninvasive indices which may be easily obtained with echocardiography and are more specific such as the longitudinal strain and the cardiac power output (CPO) have been underused or even ignored [27].

### 4.1. Left Ventricular Ejection Fraction

LV function may seem easy to evaluate, and most physicians are familiar with effortless obtained parameters such as the LVEF, which equals the LV stroke volume (LVSV) expressed as a fraction of the LV end-diastolic volume (LVEDV) [28]. It is a misconception, however, that reduced LVEF equates to LV systolic dysfunction and preserved LVEF with diastolic dysfunction. It is the nature of LV remodeling—with or without LV dilation—that is the primary driver toward reduced versus preserved LVEF [29,30]. Mathematical models can be used for the assessment of the independent effect of individual parameters. In a mathematical analysis which allowed for a systematic examination of the separate effects of LV wall thickening and reduced longitudinal velocity on ejection fraction and stroke volume, it was shown that in the presence of LV hypertrophy with preserved external cardiac dimensions, a decrease in LV long-axis shortening would not be accompanied by a concomitant impairment of ejection fraction, despite a reduction in stroke volume [31]. Based on the above it is not surprising that advanced HF may occur at any LVEF level as previously mentioned.

### 4.2. Longitudinal Strain

LV global longitudinal strain (LV-GLS) is a simple marker that reveals the longitudinal shortening as a percentage (variation in length as a proportion to baseline length) and measures the contractile function of the LV directly and more accurately than the LVEF. This is due to the fact that longitudinal subendocardial fibers are affected first and a compensatory increase in the circumferential fiber function in the presence of longitudinal dysfunction may maintain LVEF within the normal limits [32]. According to a mathematical model each 1% LV-GLS reduction should be compensated by 0.9 mm increase in wall thickness or a reduction in LV end-diastolic volume by 6–9 mL and 0.5% increase in circumferential shortening, in order to maintain LVEF [33]. Values of LV-GLS of −20% (±2%) are considered normal [34].

LV-GLS is a major independent predictor of outcome in hospitalized HF patients and has better prognostic value than LVEF. In a study including 4172 consecutive hospitalized patients with HF the primary endpoint was 5-year all-cause mortality [35]. Patients with reduced LVEF had slightly higher mortality than those with midrange or preserved LVEF (41%, 38%, and 39%, respectively; log-rank *p* = 0.031), whereas patients with reduced strain had significantly higher mortality (severely reduced LV-GLS, 49%; moderately reduced LV-GLS, 38%; mildly reduced GLS, 34%; *p* < 0.001). In multivariable analysis, each 1% increase in LV-GLS was associated with a 5% decreased risk for mortality. In contrast, patients with moderate and severe LV-GLS reductions had higher mortality, whereas LVEF was not associated with mortality. It is noteworthy that in the same study, the correlation between LV-GLS and LVEF was moderate (r = 0.69, *p* < 0.001) and LV-GLS was distributed widely at any given LVEF level [35]. Likewise, a strong prognostic capacity of LV-GLS was documented in another study including 2,104 patients hospitalized with HF who underwent echocardiography during the index admission and at least 1 additional time during a 5-year follow-up. Each 1% increase in index admission LV-GLS was associated with 10% increased odds for HF with improved LVEF among patients with HFrEF at baseline and 7% reduced odds for HF with declined LVEF among patients with HFpEF [36]. Further, in a study sample, in which data on LV-GLS were available on 2440 individuals, LV-GLS worsened across American Heart Association stages from stage A (−19.44 [3.15%]) to stage B (−18.01 [3.46%]) to stages C/D (−15.52 [4.64%]) and was correlated with death due to cardiac causes independent of clinical and cardiac factors) [37]. The accuracy and reproducibility of the measurement of LV-GLS depend on the observer experience and quality of images. However, the intraclass correlation coefficient for the measurement of LV-GLS may be significantly better than that reported for LVEF, irrespective of image quality [38].

RV systolic function exhibits a key role in the prediction of unfavorable outcomes in HF. Conventional echocardiographic parameters such as RV fractional area change (FAC) and tricuspid annular plane systolic excursion (TAPSE), have low prognostic value due to the complexity of RV geometry and load dependency of the RV functional parameters, [39,40]. In the last years, longitudinal strain has been used for the evaluation of RV systolic function and has proved a reliable evaluator of RV systolic performance, overcoming some of the limitations of conventional echocardiographic parameters as it is less load-dependent, angle-independent, highly reproducible, and measures regional myocardial deformation [41]. From the 4-chamber view, it is possible to obtain RV global longitudinal strain (RV-GLS), which encompasses the septum, and the strain of the RV free wall (RV-fwLS). Lower limits of normality for the 6-segment RV-GLS is −20.0% for men and −20.3% for women, whereas for the 3-segment RV-GLS −22.5% for men and −23.3% for women. The RV-fwLS is 5 ± 2 strain units (%) larger in magnitude than 6-segment RV-GLS and 2 ± 4% larger in women than in men [41,42]. In a population of advanced HF patients, candidates for heart transplantation (HTx), the RV-fwLS demonstrated a good association with RV stroke work index at right heart catheterization, as opposed to TAPSE and tricuspid S’ [43]. Moreover, in a recent retrospective analysis echocardiographic RV strain proved to be superior to more invasive hemodynamic measures and clinical parameters in predicting right HF following LV assist device (LVAD) implantation [44].

### 4.3. Cardiac Power Output

**Figure 3:**
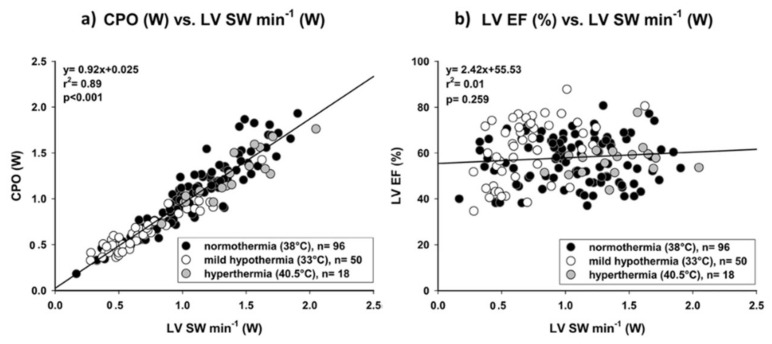
(**a**) Cardiac Power Output (CPO) accurately reflects left ventricular stroke work per minute (LV SW min^−1^) over a wide range of inotropic states. Any rise or fall of LV SW min^−1^ corresponds to an equivalent change in CPO; (**b**) Left ventricular ejection fraction (LVEF) did not correlate left ventricular stroke work per minute (LV SW min^−1^). Adapted with permission from Ref. [48]. Copyright © 2022, The Author(s). Published by Springer Nature.

LV cardiac power output (LV-CPO), which corresponds to the energy transferred from the LV to the aorta, is determined by the equation: LV-CPO (Watts or Watts/m^2^) = [cardiac output (L/min) or cardiac index (L/min/m^2^) * mean arterial blood pressure (mmHg)] * k (k = conversion factor = 1/451). LV-CPO integrates pressure (afterload), flow and heart rate (chronotropy) [45]. By coupling both pressure and flow domains of the cardiovascular system, LV-CPO is a measure of cardiac pumping. The normal resting LV-CPO is approximately 0.5–0.7 W/m^2^ and more than triples with stress (maximum LV-CPO) [46,47]. In an experimental study there was an excellent correlation between LV-CPO determined with echocardiography and the LV stroke work (LVSW)/min (Watts) determined from the pressure volume loop obtained with a conductance catheter (gold standard) as well as a lack of correlation between the LVEF and LVSW/min over a wide range of inotropic states, providing further evidence that the LVEF is a poor metric of LV systolic function (Figure 3) [48].

Under the LV-CPO umbrella the LV-CPO reserve (maximum LV-CPO-resting LV-CPO) is also included. Moreover, some studies have demonstrated that indexing LV-CPO to LV mass may be a better measure of myocardial function [49,50]. Resting LV-CPO, maximum LV-CPO, and LV-CPO reserve have been thoroughly studied and considered to predict outcomes in patients with congestive HF [46,51,52] and cardiogenic shock [53,54] as well as to follow the response to mechanical circulatory support [55,56]. Importantly, a recent study including HFpEF patients demonstrated that CPO was independently and incrementally correlated with unfavorable effects whereas other parameters of heart function such as LV size, arterial elastance, end-systolic elastance, arterial elastance/end-systolic elastance ratio, and LV mechanical efficiency were not [57].

The RV in pulmonary hypertension initially adapts to the increasing vascular load by enhancing contractility (“coupling”) to preserve flow, whereas during the late phase ventricular dilation develops in an attempt to limit the decrease in stroke volume, with uncoupling and increased wall stress as a consequence [58]. It is therefore not surprising that in a recent study including 172 patients with advanced HF, an increased RV-CPO (>0.15 Watts) calculated from the equation RV-CPO = CO [L/min] * mean pulmonary artery pressure [mmHg]/451, was independently associated with mortality [59].

## 5. Treatment

**Figure 4:**
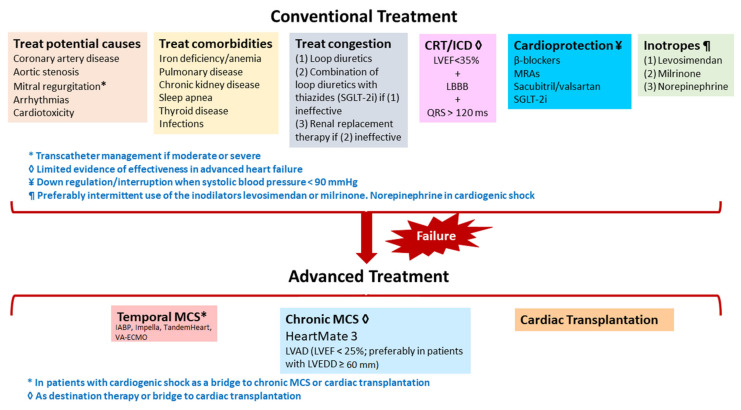
Summary of conventional and advanced treatment of advanced heart failure.

Treatment of advanced HF may be divided into conventional and advanced (Figure 4).

### 5.1. Conventional Treatment

1. **General measures.** The treatment of underlying causes should be eradicated if possible (e.g., revascularization in ischemic heart disease or aortic valve replacement in severe aortic stenosis) and comorbidities treated (e.g., supplemental intravenous iron in iron deficiency with or without iron deficiency anemia) when feasible [60].

2. **Medical treatment.** Treatment of congestion is of the upmost importance but is frequently challenging as diuretic resistance is a characteristic feature of advanced HF. Diuretic resistance implies a failure to increase fluid and sodium (Na+) output sufficiently to relieve volume overload, edema, or congestion, despite escalating doses of a loop diuretic to a ceiling level (80 mg of furosemide once or twice daily or greater in those with reduced glomerular filtration rate) [61]. The pathophysiology of diuretic resistance in HF is complicated and incompletely understood, but it is associated with several factors including renal disease, the activation of the renin-angiotensin-aldosterone system and the escape mechanisms in the kidney such as the “braking phenomenon” [62]. Additionally, other mechanisms such as the impaired intestinal absorption of oral diuretics due to the intestinal wall edema or hypoalbuminemia may be involved [63,64]. Independent predictors of diuretic resistance include oral dose of furosemide before admission and change in NT-proBNP during admission [65]. Various strategies have been successfully used to alleviate diuretic resistance, including dose escalation and continuous loop-diuretic infusion as well as diuretic combinations [66]. Multinephron segment diuretic therapy (MSDT) including concomitant use of four diuretic classes (carbonic anhydrase inhibitor, loop diuretic, thiazide, and mineralocorticoid receptor antagonist) has also been proposed [67]. Ultimately, renal replacement therapy may be used to relieve refractory congestion [14].

Hemodynamic instability often ensues as a result of cardioprotective treatment with neurohumoral inhibitors given on top of severe LV dysfunction or even severe LV dysfunction per se necessitating medication downregulation or even interruption. Eventually cardiogenic shock may develop. Ambulatory inotropic support in patients with hemodynamic instability, either with continuous infusion or intermittent inotropic therapy (IIT) of cyclic adenosine monophosphate (cAMP) dependent agents (e.g., milrinone and dobutamine) and/or calcium sensitizers (e.g., levosimendan) is utilized in ambulatory advanced HF patients for palliative care or as a “bridge” to HTx and LVAD implantation) [5,68,69]. IIT has gained popularity, especially with the use of levosimendan which has a hemodynamic effect lasting > 7 days after a 12–24 h infusion and seems to be associated with significant relief of HF symptoms and an improvement of hemodynamic, functional, and neurohormonal parameters [70]. Moreover, in a recent small study of ambulatory advanced HF patients, improvements in RV systolic function, maximal O_2_ consumption, and BNP were observed after switching from milrinone to levosimendan based IIT [71]. It is noteworthy some recent small studies suggest that treatment with the inodilators levosimendan and milrinone may also be effective in advanced HFpEF [72,73]. Hypotheses that explain the beneficial effects of inodilators in advanced HFpEF include (a) increase in the frequently reduced GLS, (b) RV function improvement, particularly in the group with pulmonary hypertension, and (c) improved LA mechanical function [74]. Norepinephrine plus dobutamine or levosimendan are indicated in patients with cardiogenic shock [69].

3. **Device treatment.** Cardiac resynchronization therapy (CRT) is indicated in HF patients in NYHA class II or III that remain symptomatic despite optimal medical treatment and have a LVEF ≤ 35% and a QRS width > 130 ms or ≥150 ms depending on the presence or absence of left bundle branch block (LBBB), respectively [75]. The potential additional placement of an implantable cardioverter-defibrillator (ICD) on CRT (CRT-D) depends on factors unrelated to LVEF such as age, extent of myocardial fibrosis, the presence or absence of coronary artery disease, life expectancy, comorbidity burden, and patients’ preference [76]. It is doubtful, however, whether the aforementioned device treatment helps in advanced HF. Indeed, absence of benefit and potential harm was reported from the National Cardiovascular Data Registry ICD Registry, which included 3343 (4.1%) patients with advanced HF and 19, 424 (23.8%) patients with non-advanced HF [77]. Both groups had received a new ICD or CRT-D implant for primary prevention of sudden cardiac death. Compared to patients with non-advanced HF, patients with advanced HF experienced clinically important periprocedural complication rates associated with in-hospital death and cardiac arrest. It was concluded that, given the observed safety concerns, the poor long-term prognosis (risk 22% for all-cause mortality at one year), and the increased competing risk of death from pump failure in patients hospitalized for HF, future randomized clinical trials should be conducted to test the safety and efficacy of the ICD and the CRT-D in patients with advanced HF [77].

### 5.2. Advanced Treatment

Patients with advanced HF and hemodynamic instability are candidates for temporary mechanical circulatory support (MCS), chronic MCS including myocardial recovery, or heart transplantation (see below).

**Figure 5:**
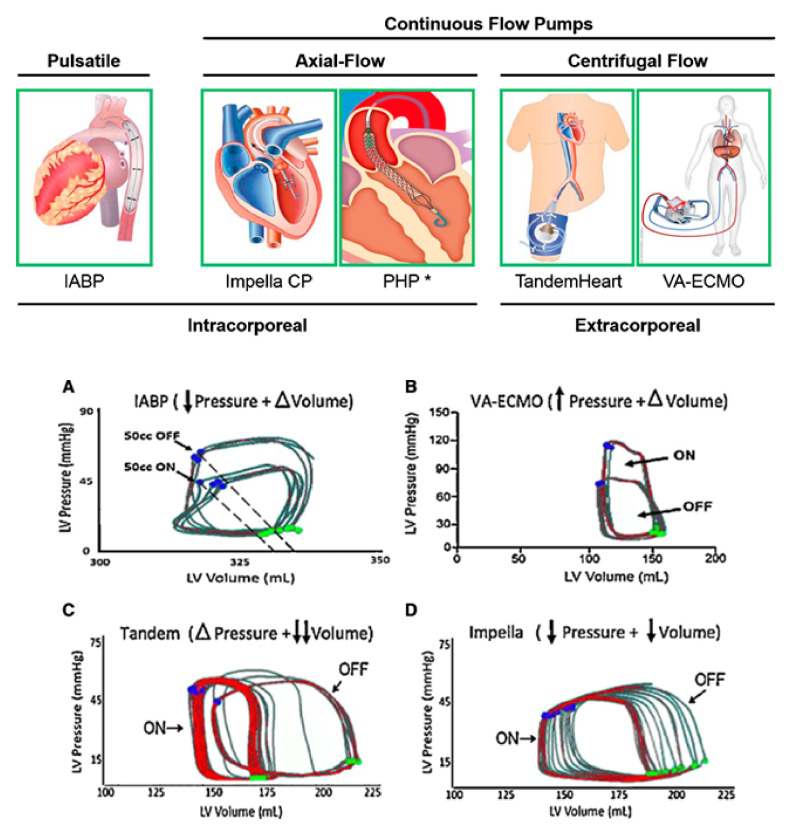
Acute mechanical circulatory support devices for the left ventricle. Pressure volume loops demonstrating hemodynamic effects of acute mechanical circulatory support devices on the left ventricle. (**A**) IABP reduces LV afterload but does not unload the ventricle. (**B**) VA-ECMO increases the wall stress and afterload of the LV and does not unload without an LV vent (**C**) The LA-FA bypass, or TandemHeart device, unloads the left atrium, thereby decreasing LV end-diastolic volumes. (**D**) The Impella device unloads the LV by decreasing end-diastolic volume and pressure. PHP= HeartMate Percutaneous Heart Pump (St. Jude Inc). * Investigational. Adapted with permission from Ref. [81]. Copyright © 2022. Published by Elsevier B.V. on behalf of Cardiological Society of India.

**Figure 6:**
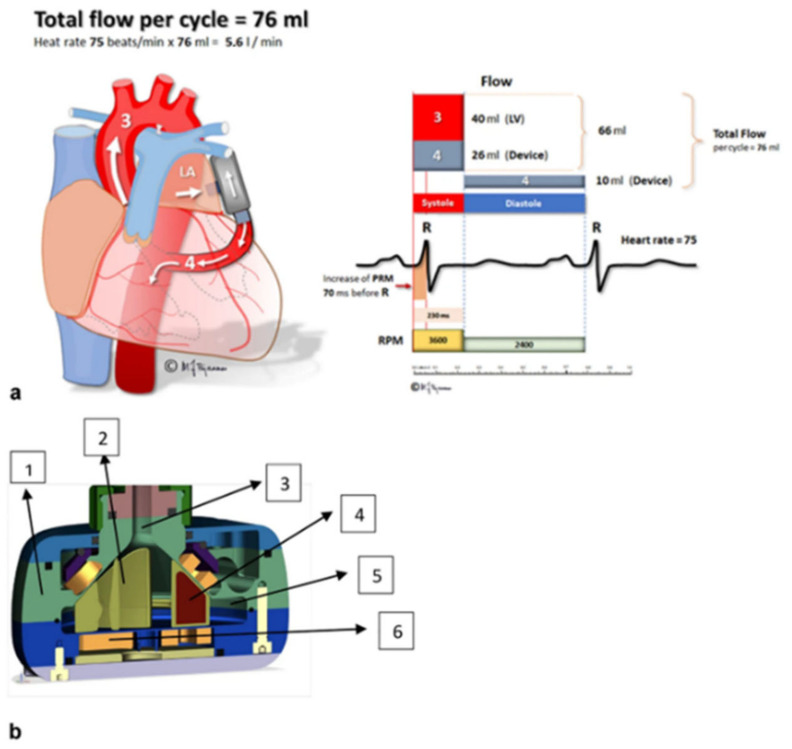
(**a**) Physiologic principle of unloading the left atrium to descending aorta. Increase in pump speed during systole to overcome the gradient from left atrium to aorta. (**b**) Cross-sectional graphic presentation of the PulseVAD. 1. Titanium casing. 2. Hydrodynamically suspended rotor. 3. Inlet tract. 4. Rare earth magnets. 5. Outlet tract. 6. Motor coils. Adapted with permission from Ref. [90]. Copyright © 2022, The Author(s). Published by Springer Nature.

1. Short-term (temporary) MCS. Although vasopressors increase mean arterial pressure and may sustain hemodynamic status in advanced HF and cardiogenic shock, their use can impair microvascular organ perfusion, increase LV afterload and myocardial work, and cause myocardial ischemia [78]. As a result, more aggressive strategies, such as temporary MCS systems, have been developed to address these issues and achieve an optimal hemodynamic status. Temporary MCS systems are percutaneous and paracorporeal devices that can be used in patients with advanced HF and cardiogenic shock for a few days, up to several weeks, in order to allow for organ recovery (cardiac, renal, liver, and brain) (Figure 5) [79,80,81]. In case of lack of organ recovery after a reasonable period of temporary MCS this treatment modality can be used as a bridge-to-decision for chronic MCS or HTx. The use of temporary MCS, which has increased substantially in recent years, despite cost, complications, and lack of high-quality data to support their use, requires clinical judgment and local expertise as the selection of the appropriate device is of the upmost importance [82].

2. Long-term (durable) MCS. LVAD are indicated in inotrope-dependent heart failure (HF) patients with pure or predominant LV dysfunction. The survival benefits are less evident in ambulatory, advanced HF [83]. Timing is crucial as early, unnecessary exposure to the risks of surgery, and device-related complications (infections, stroke, and bleeding) should be weighed against the probability of dying or developing irreversible RV and/or end-organ dysfunction while deferring implant. It is widely accepted that durable MCS should be considered following exclusion of reversible causes in patients with New York Heart Association functional class IIIB–IV, LVEF ≤ 25%, and at least one of the following criteria: (a) Interagency Registry for Mechanically Assisted Circulatory Support (INTERMACS) 2–4, (b) inotrope dependence, (c) progressive end-organ dysfunction, (d) peak VO2 <12 mL/kg/min, and c) temporary MCS dependence [84]. However, besides LVEF, LV size is independently associated with outcome. Kawabori et al. analyzed a cohort of HeartMate II recipients (n = 393) and reported that a preoperative LV end-diastolic diameter (LVEDD) < 6 cm was associated with a decreased overall survival and increased postoperative stroke incidence [85]. Recently, Truong et al. analyzed the INTERMACS registry and showed that LVEDD was an independent prognostic marker of adverse events and mortality in a cohort of cf-LVAD patients (n = 3304) which, however, mostly included patients implanted with a device recently retired from the market [86]. Finally, Molina et al. investigated the effects of LV size in a cohort of patients (n = 313) mainly implanted with a fully magnetically levitated LVAD and reported that a LVEDD cut-off point of 59 mm was associated with a worse survival and that a smaller LVEDD was an independent marker of mortality [87]. The poor outcomes of LVAD recipients with small sized LV have been attributed to technical issues [88] as well as increased platelet thrombogenicity [89]. The same concerns have been raised for the small sized ventricles of HFpEF patients necessitating alternative and potentially more technically difficult anatomical entry/inflow sites than the LV, with less robust anatomic structures and lower pressures than in the LV (Figure 6) [90]. Thus, determination of LV size is of the upmost importance prior to MCS implementation.

One of the most significant causes of postoperative morbidity and mortality post-LVAD implantation is RV failure which is estimated to manifest in 9% to 42% of the patients, based on the criteria used for the diagnosis [91]. Once biventricular support is required, 1-year survival is portended to be <50% [91,92,93]. The overall performance of existing validated models for RV failure risk prediction is undesirable, and their current clinical use remains limited. The European Registry for Patients with Mechanical Circulatory Support score seems currently to be the best model for RV failure prediction post-LVAD implantation [94].

3. Myocardial Recovery. The majority of patients with an LVAD remain on durable MCS until HTx or death. LVAD withdrawal with ventricular recovery represents the optimal outcome for patients previously implanted with an LVAD but is rare. In the INTERMACS registry (n = 13,454) the percentage of device explanted due to myocardial recovery were 0.9% at 1-year, 1.9% at 2-year, and 3.1% at 3-year follow-up [95]. As the heart recovers, it generates progressively higher systolic pressure, to the degree of overcoming the aortic pressure, leading to the aortic valve opening on every cardiac systole [96]. Independent prognostic markers of device explantation for recovery seem to be age < 50 years, nonischemic etiology, time since initial diagnosis < 2 years, suboptimal HF management before implant, LVEDD < 6.5 cm, pulmonary systolic artery pressure < 50 mm Hg, blood urea nitrogen < 30 mg/dL, and axial-flow device. Patients with myocarditis, postpartum cardiomyopathy, and Adriamycin-induced cardiomyopathy exhibit highest rates of device explantation for recovery [95].

The explant rates in more recent studies are higher and this has been attributed to the availability of percutaneous or minimally invasive short-term MCS. In the Remission from Stage D Heart Failure (RESTAGE-HF) study 40 chronic advanced HF patients with nonischemic cardiomyopathy receiving the Heartmate II LVAD were enrolled [97]. LVAD speed was optimized with an aggressive pharmacological regimen, and regular echocardiograms were performed at reduced LVAD speed (6000 rpm, no net flow) to test underlying myocardial function. Obligatory criteria were LVEDD < 60 mm, LVESD < 50 mm, LVEF > 45%, PCWP ≤ 15 mm Hg and resting cardiac index (CI) > 2.4 L/min/m^2^, whereas maximal oxygen consumption with exercise >16 mL/kg/min was an optional criterion. The primary end point of the study was the proportion of patients with sufficient improvement of myocardial function to reach criteria for explantation within 18 months with subsequent freedom from transplant/ventricular assist device/death at 12 months. 19 patients were explanted (19/36, 52.3% of those receiving the protocol). The 15 ongoing explanted patients are now 2.26 ± 0.97 years after explant. Survival free from LVAD or transplantation (after LVAD explantation) was 90% at 1-year and 77% at 2 and 3 years [97]. In another recent study 26 patients (17 HeartMate II, 9 HeartWare, 24 nonischemic etiology) underwent LVAD explantation after a median 317 days of support [98]. Prior to explantation LVAD patients were in NYHA class I, had LVEF > 40%, a cardiac index > 2.4 L/min/m^2^ and a peak oxygen intake > 50% of predicted, and had successfully undergone a 4-phase weaning assessment. At 1 year, Kaplan–Meier estimated survival was 88%, whereas at 6 years, it was 77% [98].

Major limitations of the current studies on LVAD explantation include the small number of patients and the absence of comparison data between successfully explanted and not-explanted patients, which could have shed more light on the eligibility criteria. Further, in all of these studies in which evaluation of LV systolic function is critical, the LVEF used for this purpose is affected by a multitude of factors including LV size. It is erroneous, therefore, that in a recent study evaluating the effect of LVAD on LV function, a LVEF cut-off of 40% and a LVID of 6 cm were used to classify response to LVAD [99], considering the fact that that LVID is a major LVEF determinant with LVEF decreasing when LVID increases [100]. To circumvent these limitations, it was recently proposed that LV hemodynamics of peak LV dP/dt and tau (t) should be obtained in order to define the optimal level of LVAD support in relation to LV recovery [101]. Finally, it should be mentioned that the characteristics of patients likely to recover on LVAD support are similar to those of patients who may recover spontaneously even without LVAD support. The likelihood, therefore, of myocardial recovery is to a great extend predetermined by the underlying pathophysiology (e.g., etiology and duration of HF), and the LVAD in this setting serves to ensure survival and potentially hasten recovery through ventricular unloading, maintaining end-organ function, reducing neurohormonal activation, and allowing administration of medications (e.g., β-blockers, angiotensin converting enzyme [ACE] inhibitors, or angiotensin converting blockers [ARB]) known to contribute to reverse remodeling [102].

4. Heart transplantation. Heart transplantation (HTx) is considered the “gold-standard” treatment in advanced HF. However, it has been restricted by donor availability, despite expansion of the organ pool by the greater willingness to accept marginal hearts (such as those from older donors with medical comorbidities), and use of hearts from donors who died of a drug overdose, donors with hepatitis C viremia, and donors after circulatory death [103,104]. Moreover, recently, there have been significant advances in organ allocation, donor-recipient matching, and organ preservation [105].

One principal alteration to the new allocation system was the breakdown of the previous single highest urgency status (1A) into 3 separately ranked statuses deemed to be in descending order of sickness (new status 1, 2, and 3) (Table 3) [106,107]. In this system, patients in cardiogenic shock and supported with therapy such as ECMO or other non-dischargeable biventricular mechanical circulatory support were assigned to the highest urgency status, whereas those with lesser degrees of support were distributed into a descending rank order of priority in urgency. Early investigations of new HTx allocation system suggest that revisions have resulted in broader sharing, greater ischemic times, and greater use of temporary MCS devices and have reduced the time spent waiting before receiving a transplant. Importantly, waiting list survival is improved and post-transplant outcomes are not statistically different in the most recent unadjusted Organ Procurement and Transplantation Network analysis [107].

Advances in donor organ selection, considering factors such as sex, weight, and allosensitization, offer more effective matching of donors and recipients. Sex-mismatch has been recognized as a risk marker for the most unfavorable prognosis, especially in male recipients of female heart. Although this has been ascribed to dissimilarities in the cardiovascular system between women and men, interfering variables as age, urgent transplantation, and size-mismatch should also be taken into account [108]. Donor weight < 70% of recipient weight increases mortality in non-obese heart transplant recipients, but not in obese transplant recipients [109]. Antibody-mediated rejection (AMR) is a major risk factor of impaired long-term survival after HTx. The presence of circulating donor-specific antihuman leukocyte antigen (HLA) antibodies (DSA) is considered as a mandatory criterion for antibody-mediated rejection (AMR) after HTx. DSA are known prognostic biomarkers of outcome. While desensitization protocols are typically implemented uniformly the heterogeneity in success and post-transplant outcomes argues for a more tailored approach [110]. Innovations in organ preservation such as Ex vivo machine perfusion have widened the donor pool geographically [111].

Cardiac xenotransplantation might be a promising approach to bridge the gap between the supply and demand of a donor heart [112]. Striking recent progress to avoid thrombotic microangiopathy and consumptive coagulopathy has derived from addressing the pathophysiology of these phenomena, primarily by genetic engineering of the pig to reduce graft antigenicity and to correct molecular incompatibilities in cross-species regulation of complement and coagulation pathway activation [113].

## 6. Conclusions

Advanced HF may occur at any level of LVEF, which reflects LV size rather than LV function in this setting. The introduction of specific indices for the evaluation of ventricular systolic function, such as ventricular strain and CPO, is of the upmost importance in advanced HF as these indices are better predictors of outcome than the LVEF. Undoubtedly LVEF has been used for years for the guidance of both conventional and advanced treatment in HF. However, treatment with neurohormonal inhibitors, which may be effective over a wide range of LVEF, may not be tolerated in advanced HF due to hemodynamic instability. Likewise, there is evidence to suggest that device treatment (i.e., CRT) is of limited usefulness in advanced HF. Regarding advanced treatment, mechanical circulatory support is more effective in bigger than in smaller LV due to technical issues and, therefore, the indices of LV size should be determined in all candidates for this treatment modality. In this regard, as the prevalence HFpEF is increasing in number in the aging population of the Western world, the obstacle of the small LV in this patient population can be overcome by developing adaptive MCS devices that unload the left atrium. As HTx is effective regardless of LV size, the development of technics to increase the donor pool represents a major challenge in the management of advanced HF.

## Figures and Tables

**Table 1:** Advanced Heart failure.

Characteristic	Comment	Clarifications
Admissions in hospital	Planned or unplanned	≥2 Hospitalizations or emergency department stays for decompensated HF in 12 months
Biomarkers	High risk profile	Hyponatremia Very elevated natriuretic peptides Troponin
Cardiac dysfunction	Severe structural and functional abnormalities	*Ventricular dysfunction* LV hypertrophy (concentric or eccentric) LV systolic dysfunction (abnormal global longitudinal strain and cardiac power) LV diastolic dysfunction (grade III or IV) RV dysfunction/failure (abnormal longitudinal strain and cardiac power) High risk echocardiographic features -Pulmonary hypertension -Severe mitral regurgitation refractory to decongestion *Rhythm disturbances* Arrhythmias (atrial fibrillation, ventricular tachycardia, ICD shocks) *Usual underlying causes* Ischemic heart disease Cardiomyopathy Valvular heart disease Congenital heart disease
Diuretic resistance	Common cause of recurrent rehospitalizations and death predictor	Escalating doses of diuretics (80 mg of furosemide once or twice daily or greater) Diuretic combinations Persistent edema despite escalating diuretic doses
Extracardiac organ dysfunction	When present may affect treatment options and survival	Progressive renal failure with rising creatinine/BUN Cachexia Liver dysfunction Kidney dysfunction
Functional capacity	Severe limitation	Refractory NYHA class III-IV symptoms (dyspnea, fatigue, confusion) Inability to exercise or low 6MWT (<300 m) or pVO_2_ (<12–14 mL/kg/min) Episodes of pulmonary or systemic congestion
GMT intolerance or non-response	Inability to implement appropriate treatment (dose or drug) or treatment failure	Down-titration of medical treatment as a result of hemodynamic intolerance such as hypotension (SBP < 90 mm Hg), dizziness, excessive fatigue, or nausea Discontinuation of BBs, ACE inhibitor/ARB/ARNI because of hypotension or renal intolerance Nonresponse to cardiac resynchronization therapy
Hemodynamic instability	Hypotension (SBP < 90 mmHg) with minimal or in the absence of medical treatment	Inotrope dependence Percutaneous temporary circulatory support device

LV, left ventricular; ICD, Implantable Cardioverter Defibrillator; BUN, Blood Urea Nitrogen; NYHA, New York Heart Association; 6MWT, 6-Minute Walk Test; GMT, Guideline Directed Medical Treatment; SBP, Systolic Blood Pressure; BBs. Beta-Blockers; ACE, angiotensin Converting Enzyme; ARB, Angiotensin Receptor Blockers; ARNI, Angiotensin Receptor-Neprilysin Inhibitor. Based on: Refs. [4,5].

**Table 2:** Interagency Registry for Mechanically Assisted Circulation (INTERMACS) classification system of advanced heart failure. Adapted with permission from Ref. [6]. Copyright © 2022 International Society for Heart and Lung Transplantation. Published by Elsevier Inc.

Profile	Characteristics	Risk
**Profile 1**	Cardiogenic hock	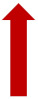
**Profile 2**	Progressive decline	
**Profile 3**	Stable, but Inotrope dependent	
**Profile 4**	Resting symptoms	
**Profile 5**	Exertion intolerant	
**Profile 6**	Exertion limited	
**Profile 7**	Advanced NYHA Class III	

**Table 3:** Comparison of old and new allocation systems.

Old Adult Allocation System	New Adult Allocation System	Criteria
**Status 1A**	**Status 1**	ECMO Non-dischargeable BiVAD MCS with VT
	**Status 2**	IABP Percutaneous VAD Surgical non-dischargeable LVAD TAH MCS with device failure VT/VF
	**Status 3**	LVAD X 30 (discretionary use) High dose or > 1 inotrope Status 1 and 2 after 14 days MCS with other complication
**Status 1B**	**Status 4**	Stable LVAD Inotropes without monitoring Retransplant
**Status 2**		Diagnosis Complex CHD HCM RCM ICM with intractable angina Amyloidosis
	**Stage 5**	Combined
	**Stage 6**	All others

ECMO, Extracorporeal Membrane Oxygenation; BiVAD, Biventricular Assist Device; MCS, Mechanical Circulatory Support; VT, Ventricular Tachycardia; IABP, Intra-aortic Balloon Pump; VAD, Ventricular Assist Device; LVAD, Left Ventricular Assist Device; TAH, Total Artificial Heart; VF, Ventricular Fibrillation; CHD, Congenital Heart Disease; HCM, Hypertrophic Cardiomyopathy; RCM, Restrictive Cardiomyopathy; ICM, Ischemic Cardiomyopathy. Adapted with permission from Ref. [107]. Copyright © 2022 by The American Association for Thoracic Surgery. Published by Elsevier.
